# *De novo* missense variants of *UNC13A* are implicated in epileptic encephalopathies and neurodevelopmental disorders

**DOI:** 10.1016/j.gendis.2024.101315

**Published:** 2024-05-06

**Authors:** Ke Su, Yu Ma, Mingshan Zhou, Yihan Liu, Chengjie Li, Yonghui Jiang, Qihui Wu, Gang Peng, Yi Wang, Shaohua Fan

**Affiliations:** a Division of Neurology, Children's Hospital of Fudan University, State Key Laboratory of Genetic Engineering, Human Phenome Institute and School of Life Sciences, Institute of Brain Science of Fudan University, Shanghai 200438, China; b Translational Research Institute of Brain and Brain-Like Intelligence, Shanghai Fourth People's Hospital Affiliated to Tongji University School of Medicine, Shanghai 200434, China; c Department of Genetics, Neuroscience, and Pediatrics, Yale University School of Medicine, New Haven, CT 06510, USA

Epilepsy is a prevalent and serious neurological disorder affecting more than 65 million individuals worldwide. The etiology of epilepsy is multifaceted, with genetic factors implicated in 70%–80% of epilepsy cases, based on early twin or family-based studies. Despite over 1000 monogenic epilepsy-associated genes have been identified, the etiology for over 50% of epilepsy cases with suspected genetic risk remains undetermined in both clinical and research studies.^1^ *UNC13A*, a gene encoding the presynaptic protein Munc13-1, plays a crucial role in neurotransmitter release at synapses.^2^ Although *UNC13A* variants have been reported to be associated with various neurological disorders,^3^ their involvement in epilepsy remains uncertain.

In the present study, we identified three *de novo* heterozygous missense variants in *UNC13A* (c.1892T>A/p.Met631Lys, c.1945T>C/p.Phe649Leu, and c.2441C>T/p.Pro814Leu in NM_001080421.3) in three unrelated probands with epileptic encephalopathies and intellectual disability (Fig. 1A; Fig. S1) based on exome sequencing of trios.

**Figure 1:**
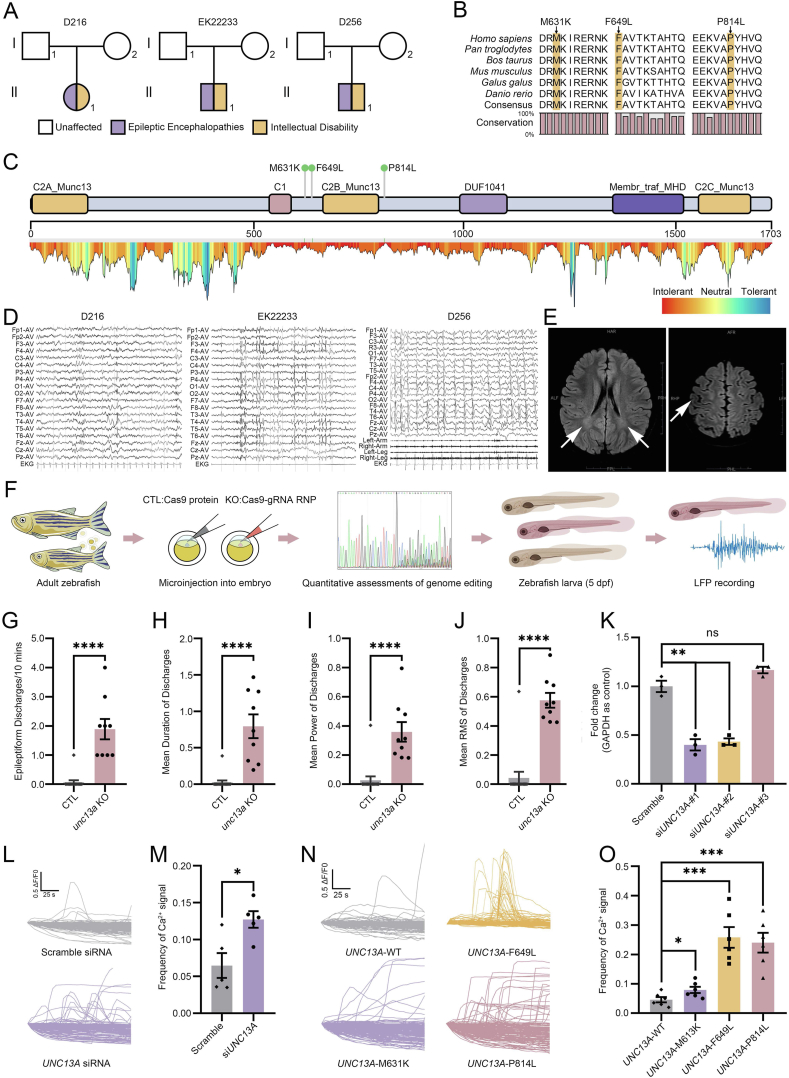
Genetic and functional analysis of three *de novo UNC13A* variants, and clinical data of the three unrelated patients with epileptic encephalopathies and intellectual disability. **(A)** Pedigrees of three patients. **(B)** Alignments show the conservation of substituted residues from zebrafish to human. Orthologs include *Homo sapiens* (NP_001073890), *Pan troglodytes* (XP_024207178), *Bos taurus* (XP_024850563), *Mus musculus* (NP_001025044), *Gallus gallus* (XP_015155241), and *Danio rerio* (NP_001038630). **(C)** Top: Munc13-1 protein schematic; arrowheads indicate variants (c.1892T>A/p.Met631Lys, c.1945T>C/p.Phe649Leu, and c.2441C>T/p.Pro814Leu in NM_001080421.3). Bottom: Intolerance landscape at UNC13A; blue (tolerant) to red (intolerant) based on MetaDome database that infers variant tolerance at each position in a human protein. **(D)** Electroencephalogram of D216_C1, EK22233_C1, and D256_C1 showed focal epilepsy discharge in the Rolandic region. **(E)** Brain magnetic resonance imaging of EK22233_C1 showed bilateral abnormal signals (indicated by white arrows) in the lateral ventricle trigone (left) and an abnormal local gyri structure in the right anterior central gyri (right). H, head (superior); F, feet (inferior); L, left; R, right; A, anterior; P, posterior. **(F)** CRISPR-Cas9 mediated mutagenesis in F0 zebrafish. We co-injected three gRNA-Cas9 ribonucleoprotein combinations targeting *unc13a* into embryos; controls received Cas9 only. **(G)** Median discharge number per 10 min in *unc13a* knockouts (*n* = 9) versus controls (*n* = 15); median: 0 [control] *vs*. 2 [knockout] per 10 min; *p <* 0.0001, two-tailed Mann–Whitney test. **(H)** Mean discharge duration (s) in knockouts versus controls; median: 0 s [control] *vs*. 0.7329 s [knockout]; *p <* 0.0001, two-tailed Mann–Whitney test. **(I)** Mean discharge power in knockouts versus controls; *p <* 0.0001, two-tailed Mann–Whitney test. **(J)** Mean discharge root mean square (RMS) in knockouts versus controls; *p <* 0.0001, two-tailed Mann–Whitney test. ∗∗∗∗*p <* 0.0001; CTL, control; LFP, local field potential. **(K)***UNC13A* knockdown efficiency in SH-SY5Y cells. Cells were transfected with scramble or *UNC13A* siRNAs for 3 d. Quantitative reverse transcription PCR showed si*UNC13A*-#1 and -#2 significantly reduced *UNC13A* mRNA (*n* = 3/treatment). **(L, M)***UNC13A* knockdown using si*UNC13A*-#1 significantly increased spontaneous calcium frequency compared with scramble siRNA in SH-SY5Y cells. The *X*-axis indicates the name of the transfected siRNA and the *Y*-axis is the frequency of the Ca^2+^ signal. One point represents one vision of the corresponding dish. **(N, O)** Overexpression of the *UNC13A* variants in HEK293T cells resulted in significant increases in calcium signal frequencies compared with the wild type. The *X*-axis shows the variant name and the *Y*-axis is the frequency of the Ca^2+^ signal. One point represents one vision of the corresponding dish. Unpaired two-tailed *t*-test: ∗*p* < 0.05; ∗∗*p* < 0.01; ∗∗∗*p* < 0.001; ns, not significant.

We observed that all three variants were not reported in gnomAD, ExAC, 1000 Genomes Project, ChinaMAP, or HUABIAO databases (Table S1). In addition, three variants were located in the sites that were conserved from zebrafish to human (Fig. 1B). MetaDome, a database that infers variant tolerance in human protein-coding genes, suggests all these three variants were located in the regions that were intolerant (c.1892T>A/p.Met631Lys and c.2441C>T/p.Pro814Leu) or relatively intolerant (c.1945T>C/p.Phe649Leu) to variants (Fig. 1C). All six *in silico* programs (SIFT, PolyPhen-2, Mutation Taster, Mutation Assessor, FATHMM, and PROVEAN) as well as CADD (Combined Annotation-Dependent Depletion, predicting the deleteriousness of variants throughout the human genome) and DANN (Deleterious Annotation of genetic variants using Neural Networks, a deep learning approach for annotating the pathogenicity of genetic variants) scores predicted these three variants to be deleterious (Table S1). In addition, we observed that *UNC13A* was predicted to be intolerant to loss-of-function (pLI = 1) and missense (*Z* = 5.63) variants in gnomAD.

Three probands exhibited consistent clinical phenotypes of developmental and epileptic encephalopathies, presenting with a history of status epilepticus, focal onset seizures in both febrile and afebrile states, and intellectual disability. The electroencephalogram findings were consistent with focal epilepsy discharge in the Rolandic region (Fig. 1D). Both D256_C1 and D216_C1 experienced early-onset epilepsy, with onset ages of 1 year and 6 months, and 1 year and 8 months, respectively. In contrast, the onset age for EK22233_C1 was 7 years old. The three probands also presented with distinctive physical features, such as a depressed nasal bridge (D256_C1) and a Café-au-Lait spot (5 cm × 5 cm) on the abdomen (D216_C1). While the brain magnetic resonance imaging of EK22233_C1 exhibited bilateral abnormal signals in the lateral ventricle trigone and an abnormal local gyri structure in the right anterior central gyri (Fig. 1E), D256_C1 and D216_C1 did not show any significant abnormalities. Furthermore, D216_C1 was diagnosed with comorbid attention deficit hyperactivity disorder. A comprehensive summary of the clinical features of the three probands is presented in Table S2.

To investigate the role of *UNC13A* in the pathogenesis of epilepsy, we first examined its function using CRISPR/Cas9 mutagenesis in an F0 zebrafish model, which has been established as a rapid and reliable system for screening genes associated with neurological diseases in humans (Fig. 1F). The *unc13a* F0 zebrafish knockouts exhibited normal development. We observed that *unc13a* F0 zebrafish knockouts exhibited a significantly higher frequency of synchronized epileptiform discharges (*p <* 0.0001, two-tailed Mann–Whitney test; Fig. 1G), prolonged duration of the discharges (*p <* 0.0001, two-tailed Mann–Whitney test; Fig. 1H), increased discharge power (*p <* 0.0001, two-tailed Mann–Whitney test; Fig. 1I), and root mean square of epileptic discharges (*p <* 0.0001, two-tailed Mann–Whitney test; Fig. 1J) compared with the controls. Consistently, we observed a significant increase in calcium fluctuation frequencies (*p =* 0.0152, unpaired two-tailed *t*-test) in *UNC13A* knockdown SH-SY5Y cells compared with the SH-SY5Y cells transfected with scramble siRNA, which indicates heightened cellular activity (Fig. 1K–M).

We further investigated the functional impacts of three variants in *UNC13A* based on the spontaneous calcium influx pattern measured after overexpressing the allele containing the variants. Due to the relatively high endogenous exp ression of *UNC13A* in SH-SY5Y cells (9.2 transcripts per million), which could interfere with our overexpression-based experiments, we utilized HEK293T cells, a widely used system in studies investigating the functional impacts of variants associated with neurological diseases. We observed that the overexpression of all three *UNC13A* variants in HEK293T cells caused significant increases in calcium fluctuation frequencies compared with the wild type (*p =* 0.0396 for c.1892T>A/p.Met631Lys, *p =* 0.0002 for c.1945T>C/p.Phe649Leu, *p =* 0.0002 for c.2441C>T/p.Pro814Leu, unpaired two-tailed *t*-test) (Fig. 1N and O). These findings strongly support the functional significance of these variants and further underscore their potential role in the pathogenesis of epilepsy. Taken together, our experimental results from knocking out, knocking down, and overexpressing variants of *UNC13A* indicate that dysfunction of this gene could give rise to epilepsy phenotypes.

*UNC13A*, which encodes Munc13-1 protein,^2^ has particularly high expression in the brain and pituitary (https://www.gtexportal.org/; Fig. S2). Munc13-1, a SNARE complex assembly factor, facilitates synaptic vesicle fusion by binding to synaptobrevin-2 and syntaxin-1 and collaborating with Munc18-1 to form the SNARE complex. This complex, crucial for synaptic vesicle transport and release, localizes to both vesicular and target membranes. Notably, variants in *STXBP1*, that encodes Munc18-1, cause developmental and epileptic encephalopathy 4 (a form of early infantile epileptic encephalopathy) with autosomal dominant or recessive inheritance patterns. This further supports the pathogenicity of *UNC13A* variants in epilepsy.

*UNC13A* variants have been suggested to be associated with neurological disorders such as amyotrophic lateral sclerosis, frontotemporal dementia, autism, microcephaly, cortical hyperexcitability, fatal myasthenia, dyskinetic movement disorder, developmental delay, intellectual disability, attention deficit hyperactivity disorder, and febrile seizures.^3^ Although the c.2441C>T/p.Phe814Leu variant was reported in a single case report of a boy with dyskinetic movement disorder, developmental delay, intellectual disability, autism, attention deficit hyperactivity disorder, and febrile seizures, his electroencephalogram was normal and he was not diagnosed with epilepsy.^4^ The phenotype difference between the patients in the prior and current study could be caused by various factors, including differences in genetic background, environmental factors, and stochastic events during development, that contribute to phenotypic diversity. A comparison of the clinical phenotypes between the reported case and D256_C1 can be found in Table S2.

Recently, *UNC13B*, a *UNC13A* homolog, has emerged as a potential epilepsy-associated gene.^5^ To gain further insights, we conducted a comparative analysis of patients with variants in either *UNC13A* or *UNC13B*, as summarized in Table S2. While the *UNC13A* and *UNC13B* patients show focal onset seizures, the electroencephalogram characteristics of *UNC13A* patients exhibited a higher propensity for discharges in the Rolandic region and seizures were observed in both febrile and afebrile states, occurring during waking and sleeping periods. In addition, the clinical phenotypes associated with *UNC13A* patients demonstrated a more severe prognosis compared with *UNC13B*. Firstly, all *UNC13B* patients displayed normal intelligence, whereas *UNC13A* patients exhibited varying degrees of intellectual disability. Secondly, none of the *UNC13B* patients experienced prolonged seizures, whereas most *UNC13A* patients had a history of status epilepticus. Lastly, *UNC13B* patients demonstrated a favorable treatment response, with all achieving seizure control through the administration of anti-seizure medications. In contrast, two *UNC13A* patients presented with refractory epilepsy, failing to achieve seizure control despite treatment with multiple anti-seizure medications.

Collectively, our results suggest *UNC13A* variants are associated with epileptic encephalopathies and intellectual disability. Importantly, our study emphasizes the clinical relevance of rare *UNC13A* variants in patients presenting with epileptic encephalopathies and intellectual disability, highlighting the potential benefits of incorporating *UNC13A* screening in future diagnostic workups.

## Ethics declaration

This study was approved by the ethics committee of the Children's Hospital of Fudan University (Ethics Approval Number: 2020-521). Written informed consent was obtained from the parents or legal guardians of all participants.

## Author contributions

S.F. and Y.W. conceived the study. K.S. carried out the data analysis and drafted the manuscript. Y.M. contributed to the analyses of patients' clinical data. M.Z. and G.P. conducted the zebrafish experiments. Y.L., K.S., and C.L. contributed to the cell experiments. S.F., K.S., Y.M., Y.J., G.P., Q.W., and Y.W. revised the manuscript. All authors read and approved the manuscript for publication.

## Data availability

The data that support the findings of this study are available from the corresponding authors.

## Conflict of interests

The authors declared no conflict of interests.

## Funding

This work was supported by the 10.13039/501100012166National Key Research and Development Program of China (No. 2020YFE0201600, 2021YFC2500202 to S.F.; 2018YFA0801000 to G.P.), the 10.13039/501100001809National Natural Science Foundation of China (No. 31970563, 32370686 to S.F.; 82101486 to Q.W.), the China's 10.13039/501100013314111 Project (No. B13016 to S.F.), the 10.13039/501100003399Shanghai Municipal Science and Technology Commission (China) (No. 19410741100 to S.F.), the Science and Technology Innovation Plan of Shanghai Science and Technology Commission (China) (No. 22ZR1414000 to G.P.), the Shanghai Municipal Science and Technology Major Project (No. 2018SHZDZX01 to G.P.; 2017SHZDZX01 to S.F.), ZJ Lab, and the Shanghai Center for Brain Science and Brain-Inspired Technology (China), the Shanghai Fourth People's Hospital affiliated to 10.13039/501100004204Tongji University 10.13039/100008235School of Medicine (No. sykyqd02301 to Q.W.), the Fundamental Research Funds for the Central Universities (China), the Shanghai Pujiang Program (China) (No. 21PJ1412100 to Q.W.), the Ningxia Hui Autonomous Region Key 10.13039/100006190Research and Development Project (China) (No. 2022BFH02012 to Q.W.), and the 10.13039/501100003399Science and Technology Commission of Shanghai Municipality, China (No. 23ZR1467900 to Q.W.). The funders had no role in study design, data collection and analysis, decision to publish, or preparation of the manuscript.
