# Supplementary material for: De novo missense variants of UNC13A are implicated in epileptic encephalopathies and neurodevelopmental disorders
Source: Genes Dis. 2024 May 6;12(2):101315. doi: 10.1016/j.gendis.2024.101315 (PMC11615879; doi:10.1016/j.gendis.2024.101315)
Supplement: Multimedia component 1 [file mmc1.docx]

**Supplementary Data**

**Supplementary materials and methods**

**Patient recruitment**

We recruited the subjects with a diagnosis of epilepsy at the Department of Neurology at the Children’s Hospital of Fudan University between January 1, 2016, and July 30, 2022. Inclusion criteria included: (1) age of onset of epilepsy 0-18 years; (2) epilepsy was diagnosed by a clinical neurologist according to the ILAE diagnostic criteria^1^ for epilepsy. Exclusion criteria include acquired epilepsy due to infection, tumor, trauma, or other causes. Clinical information was collected, including onset age of epilepsy, seizure types, responses to seizure treatment, electroencephalogram (EEG), magnetic resonance imaging (MRI) of the brain, developmental assessment, physical examination, and family history.

**Exome sequencing and analysis**

Genomic DNA was extracted from blood samples using standard procedures. Sequencing libraries were constructed and subjected to exome capture using either the SureSelect Human All Exon v5 kit (Agilent Technologies, Santa Clara, CA, USA) or xGenExome V2 kit (Integrated DNA Technologies, Coralville, IA, USA). The enriched libraries were sequenced on the Illumina HiSeq 2000/2500 or DNBSEQ-T7 platform in paired-end mode with 150 bp reads. For each sample, we obtained at least 88.18X mean depth of coverage ES data per sample.

Sequencing reads were filtered using Trimadap (v. r11, <https://github.com/lh3/trimadap>) to remove adaptors. Trimmed reads were aligned to the GRCh37 (hg19) human reference genome using the BWA^2^ mem algorithm. SAMtools^3^ (v. 1.9) was used to convert the alignment to sorted BAM format and remove ambiguously mapped reads (quality score <20). We implemented GATK^4^ (v. 4.0.10.1) best practices for germline short variant discovery to detect SNPs and small insertion and deletion (InDels). Briefly, HaplotypeCaller was used to call variants for each sample, followed by joint genotyping of all samples with CombineGVCFs. Variant quality score recalibration (VQSR) was performed using VariantRecalibrator. Low-quality variants (depth <10, genotype quality <20) and non-“PASS” flagged variants were excluded from downstream analyses.

We confirmed trio relationships using Plink v.1.90b6.21 with the ‘genome’ parameter based on variant calling results per trio.^5^

We performed variant annotation ANNOVAR^6^ (2020-06-08) based on NCBI RefSeq information downloaded from the UCSC Genome Browser (<http://hgdownload.cse.ucsc.edu/goldenpath/hg19/database/refGene.txt.gz>, file date:2020-08-18).

Variants were filtered if they: (1) fell outside exome probe target regions; (2) were synonymous; (3) had minor allele frequency >0.001 in gnomAD^7^ (v2.1.1), ExAC^8^ (v.0.3), or 1000 Genomes Project^9^ Phase 3; (4) had CADD^10^ (v1.3) score <10; or (5) were reported as “benign” in ClinVar database^11^ (accessed 2022-07-30). The functional impacts of the remaining variants were predicted using SIFT^12^, PolyPhen-2^13^, MutationTaster^14^, MutationAssessor^15^, FATHMM^16^, PROVEAN^17^, CADD^10^, and DANN^18^ based on ANNOVAR dbnsfp42a. We consider a variant causing a “damaging” effect if predicted to be deleterious by at least three algorithms (SIFT: deleterious; PolyPhen-2: possibly/probably damaging; MutationTaster: disease_causing_automatic/disease_causing; MutationAssessor: high/medium; FATHMM: deleterious; PROVEAN: deleterious; CADD ≥20; DANN ≥0.93). We annotated all the *UNC13A* variants based on reference transcript NM_001080421.3.

**Zebrafish strains**

The animal use protocols were approved by the Fudan University Shanghai Medical College Institution Animal Care and Use Committee (20210302-149). All animals were handled in accordance with the Fudan University Regulations on Animal Experiments.

Zebrafish were maintained according to standard protocols.^19^ The animals used in this study were from the AB strain. Zebrafish larvae were obtained from natural spawning and maintained in 14 h/10 h light/dark daily cycles at 28.5 ºC.

**Generation of F0 knockouts**

To disrupt the *unc13a* gene in the zebrafish, targeted lesions were introduced into the zebrafish genome following a published method.^20^ The target sequences of guide RNA (gRNA) in the present study were designed based on a prior study (http://skl.scau.edu.cn/targetdesign/). The off-target scores for the three gRNAs were estimated to be 0.159, 0.232, and 0.008, respectively, all of which fall below the recommended threshold of 0.6 for minimizing potential off-target effects. One-cell stage zebrafish embryos were injected with Cas9-gRNA (**Table S3**) ribonucleoprotein complexes targeting three regions of *unc13a*. Quantitative assessments of genome editing were conducted using TIDE^21^ and DECOD^22^. Sanger sequencing confirmed that frameshift variants were introduced at the targeted sites and the depletion rate is >90% in the F0 knockout zebrafishes. Control animals were injected with Cas9 protein only.

**Local field potential (LFP) recording**

The development of *unc13a* F0 knockouts proceeded normally through 5 days post-fertilization (dpf), at which point we recorded forebrain LFPs. LFP recordings were performed as described previously^23,24^ with modifications. Zebrafish larvae 5dpf were embedded in 1% low melt point agarose. A recording electrode was inserted into the dorsal forebrain and a reference electrode was placed over the hindbrain. Insulated stainless steel electrodes were used as described previously.^23^ LFPs were sampled at 5 kHz, denoised by wavelet decomposition, and epileptiform discharges were identified and quantified using custom MATLAB scripts.

**Human neuroblastoma SH-SY5Y cell-based calcium imaging**

Human neuroblastoma SH-SY5Y cells were plated on coverslips in 24-well plates. Three siRNAs (**Table S3**) targeting *UNC13A* or a scramble siRNA were transfected with pLVX-GCaMP6s plasmids using Lipofectamine 2000 (Invitrogen). Three days post-transfection, cells were imaged using an Axio Observer Z1 (Zeiss). Spontaneous calcium fluctuations were recorded at 5 s intervals (**Supplemental Video 1 and 2**). ImageJ (NIH, 1.50i) was used to quantify fluorescence, with baseline (F0) defined as the 5 s preceding each peak. Peaks were identified as ΔF/F0 > 2SD of F0. We tested the knock down efficiency of three siRNAs using real-time qPCR assay.

**Plasmids construction**

Human WT *UNC13A* was obtained by subcloning the plasmid templates into the FUGW-2*His-P2A-mCherry vector (based on the FUGW vector, modified by our lab). The missense variant c.1892T>A/p.Met631Lys, c1945T>C/p.Phe649Leu, and c.2441C>T/p.Pro814Leu was constructed by overlap PCR mutagenesis. The primers were listed in **Table S3**.

**HEK293T cell-based calcium imaging**

Both human neuroblastoma SH-SY5Y cells and HEK293T cells have been used in functional studies of epilepsy-related variants.^25-27^ Considering the substantial difference in *UNC13A* expression levels between SH-SY5Y cells (nTPM: 9.2) and HEK293T cells (nTPM: 0.1; Human Protein Atlas),^28^ we overexpressed plasmid containing wildtype or *UNC13A* variants in HEK293T cells. Ca^2+^ indicators (GCaMPs) or Ca^2+^ channel subunits (CaV1.2, CaVβ2a) have been overexpressed in HEK293T cells to study Ca^2+^ influx or Ca^2+^ currents in previous studies.^29-32^ Additionally, calcium imaging in HEK293T cells has been utilized to investigate the function of genes and variants associated with Alzheimer's disease.^33^

pLVX plasmids encoding GCaMP6s and lentiviral packaging components were transfected into HEK293T cells using Lipofectamine 2000 (Invitrogen). Lentivirus was collected 48-72 h post-transfection, purified, and concentrated. To generate a stable GCaMP6s-expressing cell line, 2 μL purified lentivirus was added to HEK293T cells, and cells were selected with puromycin. Media was refreshed every 2 days for 2 weeks until clone formation. One day before imaging, GCaMP6s-expressing HEK293T cells were plated on 35 mm Mattek dishes. Wildtype or mutant *UNC13A* constructs were transfected using Lipofectamine 2000. Two days post-transfection, cells were imaged using an Axio Observer Z1 (Zeiss) at 5 s intervals to detect spontaneous calcium influx (**Supplemental Video 3-6**). Calcium fluctuations were measured using the GCaMP6s calcium sensor in HEK293T cells.

**Statistical analysis**

Quantitative data in all bar charts are presented as mean ± standard error of the mean (SEM). Since the results of knocking down in human neuroblastoma SH-SY5Y cells and overexpression in HEK293T cells conformed to the normal distribution based on the Shapiro-Wilk test, we used unpaired two-tailed *t*-test to compare whether the differences from these experiments are significant or not. We used the two-tailed Mann-Whitney test to compare LFP differences between zebrafish knockouts and controls. Statistical analyses were performed with GraphPad Prism 8.0.1. *P* < 0.05 were considered statistically significant.

**Supplementary result**

**Drug therapy and outcomes of the three patients**

Regarding drug therapy (**Table S2**), D216_C1 achieved seizure control through levetiracetam (LEV) monotherapy, while D256_C1 and EK22233_C1 exhibited refractory epilepsy. Initially, D256_C1 experienced seizure control with LEV monotherapy but later relapsed after one year and remained uncontrolled despite receiving lacosamide (LCM), nitrazepam (NZP), and perampanel (PER). EK22233_C1 achieved seizure control for two months through the administration of PER, after unsuccessful treatment with valproic acid (VPA), LCM, and clonazepam (CLZ).

**Supplementary references**

1. Fisher RS, Acevedo C, Arzimanoglou A, et al. ILAE official report: a practical clinical definition of epilepsy. *Epilepsia*. 2014;55(4):475-82. <https://doi.org/10.1111/epi.12550>

2. Li H, Durbin R. Fast and accurate short read alignment with Burrows-Wheeler transform. *Bioinformatics*. 2009;25(14):1754-1760. <https://doi.org/10.1093/bioinformatics/btp324>

3. Li H, Handsaker B, Wysoker A, et al. The Sequence Alignment/Map format and SAMtools. *Bioinformatics*. 2009;25(16):2078-2079. <https://doi.org/10.1093/bioinformatics/btp352>

4. McKenna A, Hanna M, Banks E, et al. The Genome Analysis Toolkit: a MapReduce framework for analyzing next-generation DNA sequencing data. *Genome Res*. 2010;20(9):1297-1303. <https://doi.org/10.1101/gr.107524.110>

5. Purcell S, Neale B, Todd-Brown K, et al. PLINK: a tool set for whole-genome association and population-based linkage analyses. *Am J Hum Genet*. 2007;81(3):559-575. <https://doi.org/10.1086/519795>

6. Wang K, Li M, Hakonarson H. ANNOVAR: functional annotation of genetic variants from high-throughput sequencing data. *Nucleic Acids Res*. 2010;38(16):e164. <https://doi.org/10.1093/nar/gkq603>

7. Karczewski KJ, Francioli LC, Tiao G, et al. The mutational constraint spectrum quantified from variation in 141,456 humans. *Nature*. 2020;581(7809):434-443. <https://doi.org/10.1038/s41586-020-2308-7>

8. Lek M, Karczewski KJ, Minikel EV, et al. Analysis of protein-coding genetic variation in 60,706 humans. *Nature*. 2016;536(7616):285-291. <https://doi.org/10.1038/nature19057>

9. 1000 Genomes Project Consortium, Auton A, Brooks LD, et al. A global reference for human genetic variation. *Nature*. 2015;526(7571):68-74. <https://doi.org/10.1038/nature15393>

10. Kircher M, Witten DM, Jain P, O'Roak BJ, Cooper GM, Shendure J. A general framework for estimating the relative pathogenicity of human genetic variants. *Nat Genet*. 2014;46(3):310-5. <https://doi.org/10.1038/ng.2892>

11. Landrum MJ, Lee JM, Benson M, et al. ClinVar: improving access to variant interpretations and supporting evidence. *Nucleic Acids Res*. 2018;46(D1):D1062-D1067. <https://doi.org/10.1093/nar/gkx1153>

12. Kumar P, Henikoff S, Ng PC. Predicting the effects of coding non-synonymous variants on protein function using the SIFT algorithm. *Nat Protoc*. 2009;4(7):1073-81. <https://doi.org/10.1038/nprot.2009.86>

13. Adzhubei IA, Schmidt S, Peshkin L, et al. A method and server for predicting damaging missense mutations. *Nat Methods*. 2010;7(4):248-9. <https://doi.org/10.1038/nmeth0410-248>

14. Schwarz JM, Cooper DN, Schuelke M, Seelow D. MutationTaster2: mutation prediction for the deep-sequencing age. *Nat Methods*. 2014;11(4):361-2. <https://doi.org/10.1038/nmeth.2890>

15. Reva B, Antipin Y, Sander C. Predicting the functional impact of protein mutations: application to cancer genomics. *Nucleic Acids Res*. 2011;39(17):e118. <https://doi.org/10.1093/nar/gkr407>

16. Shihab HA, Gough J, Cooper DN, et al. Predicting the functional, molecular, and phenotypic consequences of amino acid substitutions using hidden Markov models. *Hum Mutat*. 2013;34(1):57-65. <https://doi.org/10.1002/humu.22225>

17. Choi Y, Sims GE, Murphy S, Miller JR, Chan AP. Predicting the functional effect of amino acid substitutions and indels. *PLoS One*. 2012;7(10):e46688. <https://doi.org/10.1371/journal.pone.0046688>

18. Quang D, Chen Y, Xie X. DANN: a deep learning approach for annotating the pathogenicity of genetic variants. *Bioinformatics*. 2015;31(5):761-3. <https://doi.org/10.1093/bioinformatics/btu703>

19. Westerfield M. *The zebrafish book : a guide for the laboratory use of zebrafish (Danio rerio)*. Printed by the University of Oregon Press : Distributed by the Zebrafish International Resource Center; 2007.

20. Kroll F, Powell GT, Ghosh M, et al. A simple and effective F0 knockout method for rapid screening of behaviour and other complex phenotypes. *Elife*. 2021;10<https://doi.org/10.7554/eLife.59683>

21. Brinkman EK, Chen T, Amendola M, van Steensel B. Easy quantitative assessment of genome editing by sequence trace decomposition. *Nucleic Acids Res*. 2014;42(22):e168. <https://doi.org/10.1093/nar/gku936>

22. Bloh K, Kanchana R, Bialk P, et al. Deconvolution of Complex DNA Repair (DECODR): Establishing a Novel Deconvolution Algorithm for Comprehensive Analysis of CRISPR-Edited Sanger Sequencing Data. *CRISPR J*. 2021;4(1):120-131. <https://doi.org/10.1089/crispr.2020.0022>

23. Gao G, Guo S, Zhang Q, Zhang H, Zhang C, Peng G. Kiaa1024L/Minar2 is essential for hearing by regulating cholesterol distribution in hair bundles. *Elife*. 2022;11<https://doi.org/10.7554/eLife.80865>

24. Baraban SC. Forebrain electrophysiological recording in larval zebrafish. *J Vis Exp*. 2013;(71)<https://doi.org/10.3791/50104>

25. Xu W, Zhang W, Cui L, et al. Novel mutation of SIK1 gene causing a mild form of pediatric epilepsy in a Chinese patient. *Metab Brain Dis*. 2022;37(4):1207-1219. <https://doi.org/10.1007/s11011-022-00943-4>

26. He N, Guan BZ, Wang J, et al. HCFC1 variants in the proteolysis domain are associated with X-linked idiopathic partial epilepsy: Exploring the underlying mechanism. *Clin Transl Med*. 2023;13(6):e1289. <https://doi.org/10.1002/ctm2.1289>

27. Rodan LH, Spillmann RC, Kurata HT, et al. Phenotypic expansion of CACNA1C-associated disorders to include isolated neurological manifestations. *Genet Med*. 2021;23(10):1922-1932. <https://doi.org/10.1038/s41436-021-01232-8>

28. Ponten F, Jirstrom K, Uhlen M. The Human Protein Atlas--a tool for pathology. *J Pathol*. 2008;216(4):387-93. <https://doi.org/10.1002/path.2440>

29. Toth PT, Shekter LR, Ma GH, Philipson LH, Miller RJ. Selective G-protein regulation of neuronal calcium channels. *J Neurosci*. 1996;16(15):4617-24. <https://doi.org/10.1523/JNEUROSCI.16-15-04617.1996>

30. Heim N, Griesbeck O. Genetically encoded indicators of cellular calcium dynamics based on troponin C and green fluorescent protein. *J Biol Chem*. 2004;279(14):14280-6. <https://doi.org/10.1074/jbc.M312751200>

31. Akerboom J, Carreras Calderon N, Tian L, et al. Genetically encoded calcium indicators for multi-color neural activity imaging and combination with optogenetics. *Front Mol Neurosci*. 2013;6:2. <https://doi.org/10.3389/fnmol.2013.00002>

32. Montmayeur JP, Barr TP, Kam SA, Packer SJ, Strichartz GR. ET-1 induced Elevation of intracellular calcium in clonal neuronal and embryonic kidney cells involves endogenous endothelin-A receptors linked to phospholipase C through Galpha(q/11). *Pharmacol Res*. 2011;64(3):258-67. <https://doi.org/10.1016/j.phrs.2011.04.003>

33. Honarnejad K, Jung CK, Lammich S, Arzberger T, Kretzschmar H, Herms J. Involvement of presenilin holoprotein upregulation in calcium dyshomeostasis of Alzheimer's disease. *J Cell Mol Med*. 2013;17(2):293-302. <https://doi.org/10.1111/jcmm.12008>

**Supplementary figures**

**Figure S1**


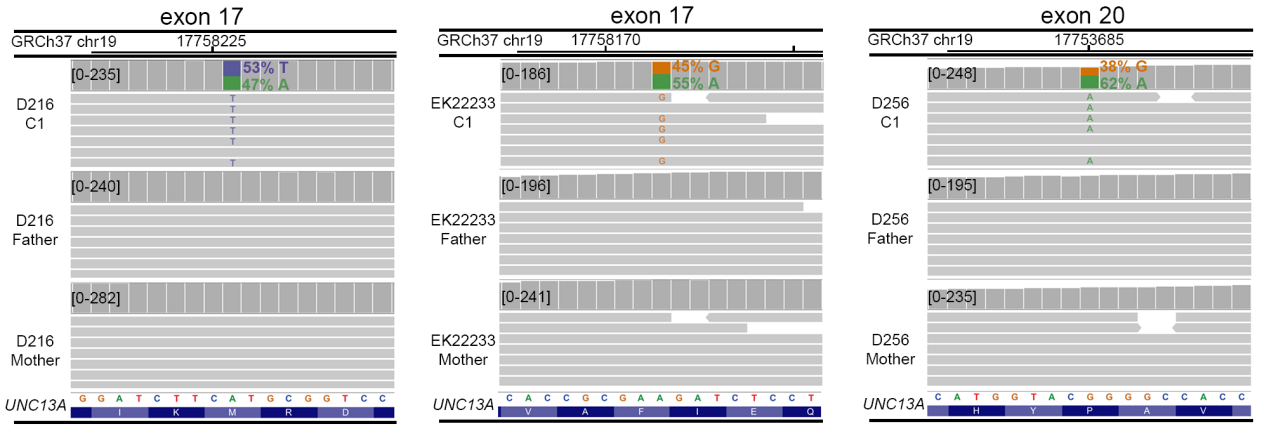


**Figure S1. Integrative Genomics Viewer visualization of depth of coverage at c.1892T>A/p.Met631Lys, c.2441C>T/p.Pro814Leu, and c1945T>C/p.Phe649Leu.** All three variants have ≥ 76 supportive reads.

**Figure S2**


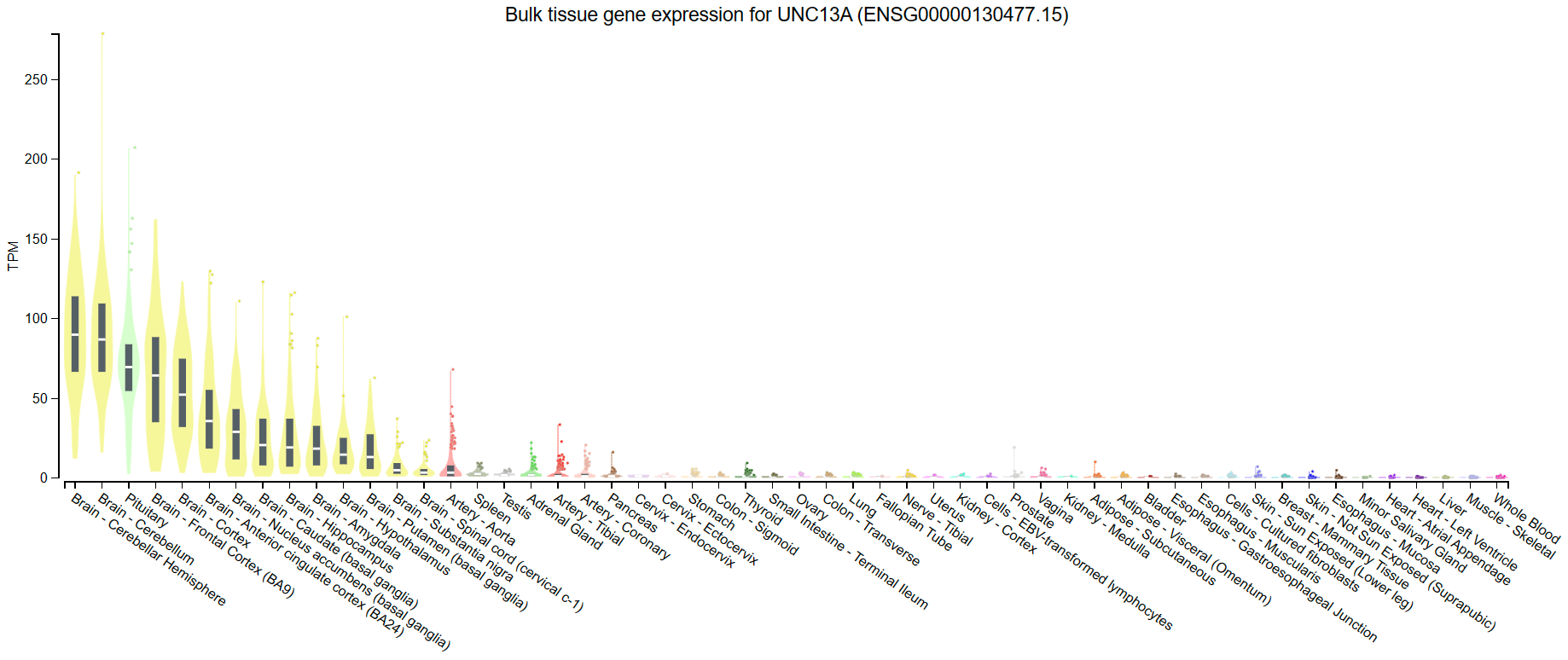


**Figure S2. RNA expression of *UNC13A* in human normal tissue plotted as transcripts per million (TPM).** The plot was obtained from the GTEx database (<https://www.gtexportal.org/>).

**Supplementary tables**

**Table S1.** Summary of the three *de novo* variants in *UNC13A* in the present study

| Patient | cDNA change *^a^* | Amino acid change | Minor allele frequency | | | | |  | In silico prediction *^b^* | | | | | | | |
| --- | --- | --- | --- | --- | --- | --- | --- | --- | --- | --- | --- | --- | --- | --- | --- | --- |
|  |  |  | **1KGP_EAS** | **gnomAD_EAS** | **ExAC_EAS** | **HUABIAO** | **ChinaMAP** |  | **SIFT** | **PolyPhen-2** | **Mutation Taster** | **Mutation Assessor** | **FATHMM** | **PROVEAN** | **CADD** | **DANN** |
| D216_C1 | c.1892T>A | p.M631K | . | . | . | . | . |  | D | P | D | M | T | D | 27.9 | 0.988 |
| EK22233_C1 | c.1945T>C | p.F649L | . | . | . | . | . |  | D | P | D | M | T | D | 30 | 0.998 |
| D256_C1 | c.2441C>T | p.P814L | . | . | . | . | . |  | D | D | D | M | T | D | 28.1 | 0.998 |

*^a^*The GenBank accession number of *UNC13A* is NM_00108042.

*^b^*D, deleterious; P, possibly damaging; M, medium; T, Tolerated. Higher CADD and DANN scores mean that the variants are more likely to be considered as deleterious variants.

“.” indicates that the variants were not reported in the 1KGP_EAS, gnomAD_EAS, ExAC_EAS, HUABIAO, and ChinaMAP datasets.

1KGP_EAS, eastern Asians in the 1000 genome project; ExAC_EAS, eastern Asians in the ExAC dataset; gnomAD_EAS, eastern Asians in the gnomAD project.

**Table S2. Clinical features of patients with *de novo* variants in *UNC13A* and a comparison between epilepsy patients with variants in *UNC13A* or *UNC13B***

| Gene | Patient | Gender | Age of onset | Variant | Inheritance | Diagnosis | FS | SE | Seizure timing | Treatment | EEG | Brain MRI | Development |
| --- | --- | --- | --- | --- | --- | --- | --- | --- | --- | --- | --- | --- | --- |
| *UNC13A* | D216_C1 | female | 1y and 8mo | c.1892T>A(p.Met631Lys) | *de novo* | PE, SE, ID | Yes | Yes | Awaking | LEV, seizure free at 4y | Spike slow wave discharge in the Rolandic region on both sides | Normal | ID, DQ<49, MI 51 |
|  | EK22233_C1 | male | 7y | c.1945T>C(p.Phe649Leu) | *de novo* | PE, SE, ID | Yes | Yes | Sleeping | Refractory to valproic acid, lacoxamine and clonazepam. Response to pirampanide, seizure free at 10y | Spike wave discharge in the Rolandic region on the right | Bilateral abnormal signals in the lateral ventricle trigone, and unnatural local gyri structure of the right anterior central gyri | ID, IQ<40 |
|  | D256_C1 | male | 1y and 6mo | c.2441C>T(p.Pho814Leu) | *de novo* | PE, SE, ID | Yes | Yes | Sleeping | Refractory to lacoxamine, nitrazepam and pirampanide | Sharp slow wave discharge in the Rolandic region on the right | Normal | ID, DQ<47,MI<47 |
|  | (Lipstein et al.4)Case | male | 4y (FS) | c.2441C>T(p.Pho814Leu) | *de novo* | Dyskinetic movement disorder  ID, ASD, ADHD, FS | Yes | No | NA | Methylphenidate for ADHD | Normal | Normal | developmental delay, nonverbal IQ of 70 |
| *UNC13B* (Wang et al.5） | 1 | female | 7y | c.135G>A (p.Trp45X) | *de novo* | PE (BOE) | No | No | Nocturnal | OXC, seizure free at 8y | Left occipital spikes and slow spike waves | Normal | Normal |
|  | 2 | male | 5y | c.4008+1G>T | From unaffected mother | PE (SeLECTS) | No | No | Mostly nocturnal | VPA, LEV, seizure free at 8y | Right central-temporal sharp and sharp-slow waves | NA | Normal |
|  | 3 | male | 12y | c.4330+7G>A | *de novo* | PE (SeLECTS) | No | No | Nocturnal | OXC, seizure free at 13y | Left, right and bilateral central-temporal spikes | Normal | Normal |
|  | 4-1 | female | 3mo | c.662G>A (p.Arg221Gln) | From FS mother | PE | No | No | Mostly on  awakening | OXC, seizure free at 3mo | Ictal: right frontal originating CPS; interictal: no discharge | Normal | Normal |
|  | 4-2 | female | 1y | c.662G>A (p.Arg221Gln) | 4-1 mother, unknown | FS | Yes | No | - | - | NA | Normal | Normal |
|  | 5-1 | female | 8mo | c.1981C>T (p.Arg661Cys) | From affected mother | PE | No | No | Mostly diurnal | LEV, seizure free at 1y | Frontal and midline sharp waves and sharp-slow waves | Normal | Normal |
|  | 5-2 | female | 3y | c.1981C>T (p.Arg661Cys) | 5-1 mother, unknown | UE | No | No | NA | - | NA | Normal | Normal |
|  | 6-1 | female | 7mo | c.2381G>A (p.Gly794Asp) | From FS father | FS, PE | Yes | No | Game-precipitated, nocturnal | LTG, VPA, seizure free at 18y | Left central-temporal small spikes | Normal | Normal |
|  | 6-2 | male | 1y | c.2381G>A (p.Gly794Asp) | 6-1 father, unknown | FS | Yes | No | - | - | NA | Normal | Normal |
|  | 7-1 | male | 2y | c.2644G>T (p.Gly882Trp) | From FS mother | FS, PE | Yes | No | Diurnal and Nocturnal | LEV, seizure free at 4y | Right frontal and central-temporal spikes and sharp waves, and sharp/slow spike waves | Normal | Normal |
|  | 7-2 | female | 2y | c.2644G>T (p.Gly882Trp) | 7-1 mother, unknown | FS | Yes | No | - | - | NA | Normal | Normal |
|  | 8 | female | 22y | c.308C>T (p.Thr103Met) c.1190C>T (p.Ser397Phe) | respectively from parents | PE | No | No | Diurnal and Nocturnal | LTG, seizure free at 28y | Ictal: 1 sGTCS and 4 CPS of indeterminate origin; interictal: left and right temporal spikes and sharp waves | Abnormal, structural asymmetry in the hippocampus | Normal |

*UNC13A*: NM_001080421.3; *UNC13B*: NM_006377.3; Abbreviations: ADHD, attention deficit hyperactivity disorder; AR, autosomal recessive; ASD, autism spectrum disorder; BOE, benign occipital epilepsy; CPS, complex partial seizure; DQ, developmental quotient; EEG, electroencephalography; FS, febrile seizure; GTCS, generalized tonic-clonic seizure; ID, intellectual disability; IQ, intelligence quotient; LEV, levetiracetam; LTG, lamotrigine; MAF, minor allele frequency; MI, mental index; MRI, magnetic resonance imaging; NA, not available; OXC, oxcarbazepine; PE, partial epilepsy; SE, status epilepticus; SeLECTS, Self-limited epilepsy with centrotemporal spikes; sGTCS, secondary generalized tonic-clonic seizure; UE, unclassified epilepsy; VPA, valproate; mo, months; y, years.

| Name | Sequence (5'-3') | Application |
| --- | --- | --- |
|  |  |  |
| *unc13a* gRNA-1 | GGAGCAGCTCAATGCCATGAGGG | disrupting the *unc13a* gene in the zebrafish |
| *unc13a* gRNA-2 | GGGATGTGGTGTAGAAAGGAGGG | disrupting the *unc13a* gene in the zebrafish |
| *unc13a* gRNA-3 | GGCCAAACTCCTCACTGAGTTGG | disrupting the *unc13a* gene in the zebrafish |
| *UNC13A*-siRNA-#1 | GGATTGACCTCTCCATGTA | knocking down *UNC13A* gene in SH-SY5Y cells |
| *UNC13A*-siRNA-#2 | GAACCCAGATGATCTTCAA | knocking down *UNC13A* gene in SH-SY5Y cells |
| *UNC13A*-siRNA-#3 | GCGGTCTCATCATCATCGA | knocking down *UNC13A* gene in SH-SY5Y cells |
| *UNC13A*-WT-F | CGACTCTAGAGGATCCGCCACCATGTCTCTGCTTTGCGTTGGAGT | PCR/Clone |
| *UNC13A*-WT-R | GGTGATGGTGGGATCCAGGCGCAGGCGCGGCACCGC | PCR/Clone |
| *UNC13A*-M631K-F | GACCGCAAGAAGATCCGGGAGCGCAACA | Point mutation |
| *UNC13A*-M631K-R | GGATCTTCTTGCGGTCCTTGAGCACCATG | Point mutation |
| *UNC13A*-F649L-F | CATCCAGGAGATCCTCGCGGTGACCAAGACGGC | Point mutation |
| *UNC13A*-F649L-R | GTCACCGCGAGGATCTCCTGGATGAGCTCGAAGATC | Point mutation |
| *UNC13A*-P814L-F | GTGGCCCTGTACCATGTCCAGTACACCTGTCT | Point mutation |
| *UNC13A*-P814L-R | ATGGTACAGGGCCACCTTCTCCTCGC | Point mutation |

**Table S3.** DNA sequences of primers or target sequences of gRNAs and siRNA
